# Editorial: Treating solid organ diseases with innovative chimeric-antigen receptor T-cells: current indications, progress, limitations and potential strategies

**DOI:** 10.3389/fimmu.2024.1491982

**Published:** 2024-09-24

**Authors:** Elizabeth Quackenbush, Jyothi Vijayaraghavan, Emrah Ilker Ozay, Rui-Ru Ji

**Affiliations:** ^1^ Consultant, New York, NY, United States; ^2^ Department of Clinical Development, Viridian Therapeutics, Inc., Waltham, MA, United States; ^3^ Department of Immunology, Orbital Therapeutics, Inc., Cambridge, MA, United States; ^4^ Department of Translational Medicine, Akamis Bio, Cambridge, MA, United States

**Keywords:** CAR-T cells, solid tumor, anti-tumor activity, persistence, tumor infiltration, therapeutic efficacy

Recently (July 2024), a CAR-T therapy (lisocabtagene maraleucel, or Breyanzi) was approved by the U.S. FDA for resistant mantle cell lymphoma (MCL). This extends the list of approvals for this revolutionary cancer treatment to six, with all approvals being restricted to hematological malignancies. In selective leukemias and lymphomas, CAR-T cells have produced remarkable responses, showing objective response rates of 80% or more. However, significant challenges limit the efficacy of CAR-T cells for solid tumors. Physical and environmental barriers to creating effective CAR-T cell therapy for solid tumor indications are broadly grouped into mechanistic categories of: 1) cell trafficking; 2) tumor metabolism; and 3) immunological regulation (1). Thus, a multi-pronged effort is needed to develop and implement innovative CAR-T cells that can improve anti-solid tumor activity and decrease toxicity. Furthermore, with the exciting new approach of using CAR-T cells as a treatment for selective auto-immune disorders (2), the need for significant improvements in efficacy is heightened. In this Research Topic, we present five articles that summarize the state of the art of CAR-T cell therapy for solid tumors and present experimental strategies to overcome some of the challenges that limit efficacy.

In their review article, Zhou et al. summarize novel engineering and pharmaceutical interventions designed to address many barriers, and they discuss the latest studies that are expected to reach the clinic in the next few years. They close by presenting future potential directions, including CRISPR-based CAR genetic modifications and the generation of CAR T cells from progenitor-like T cells.

Smirnov et al. focus on improvements in our understanding of CAR-T cell signaling. For example, they discuss how the field is now using fifth generation CAR-T cells in preclinical trials, whereas second generation CAR-T cells introduced a costimulatory domain for better long-term CAR-T cell engraftment and efficacy. They highlight how the processes that orchestrate the activation and differentiation of CAR-T cells into a specific phenotype required for long-term persistence are not fully understood, and they discuss research aimed at elucidating how CAR domains and T-cell signaling molecules are involved in these processes.

Satapathy et al., in their systematic review, analyze combination therapy involving CAR-T cells and anti PD-1 agents, an approach that is aimed at overcoming barriers of tumor inhibition, T-cell exhaustion, heightened T-cell activation, and unwanted toxicities. Topics discussed include the ability to achieve better overall response rate (ORR) and progression-free survival (PFS) in both pre-clinical and clinical models and the implications and feasibility of combination immunotherapies.

Finally, two papers present new experimental data on models that use innovative approaches. For example, Nagy et al. are working to improve the penetration of universal CAR-T cells into the extracellular matrix of ADCC-resistant tumors, using molecular tags to target a variety of different tumor antigens. In contrast, Yang et al. hypothesize that the delivery of radiation to tumors can synergize with CAR-T therapy, resulting in enhanced anti-tumor immunity and tumor response. They studied the feasibility of this approach by delivering β-emitting ^177^Lu-DOTATATE to subcutaneous tumors along with tumor-infiltrating CAR T cells expressing somatostatin receptor 2.
